# Spiking network optimized for word recognition in noise predicts auditory system hierarchy

**DOI:** 10.1371/journal.pcbi.1007558

**Published:** 2020-06-19

**Authors:** Fatemeh Khatami, Monty A. Escabí

**Affiliations:** 1 Department of Bioengineering, School of Engineering and Computer Science, University of the Pacific, Stockton, California, United States of America; 2 Department of Biomedical Engineering, University of Connecticut, Storrs, Connecticut, United States of America; 3 Department of Electrical and Computer Engineering, University of Connecticut, Storrs, Connecticut, United States of America; 4 Department of Psychological Sciences, University of Connecticut, Storrs, Connecticut, United States of America; 5 Connecticut Institute for Brain and Cognitive Sciences, University of Connecticut, Storrs, Connecticut, United States of America; University of Pennsylvania, UNITED STATES

## Abstract

The auditory neural code is resilient to acoustic variability and capable of recognizing sounds amongst competing sound sources, yet, the transformations enabling noise robust abilities are largely unknown. We report that a hierarchical spiking neural network (HSNN) optimized to maximize word recognition accuracy in noise and multiple talkers predicts organizational hierarchy of the ascending auditory pathway. Comparisons with data from auditory nerve, midbrain, thalamus and cortex reveals that the optimal HSNN predicts several transformations of the ascending auditory pathway including a sequential loss of temporal resolution and synchronization ability, increasing sparseness, and selectivity. The optimal organizational scheme enhances performance by selectively filtering out noise and fast temporal cues such as voicing periodicity, that are not directly relevant to the word recognition task. An identical network arranged to enable high information transfer fails to predict auditory pathway organization and has substantially poorer performance. Furthermore, conventional single-layer linear and nonlinear receptive field networks that capture the overall feature extraction of the HSNN fail to achieve similar performance. The findings suggest that the auditory pathway hierarchy and its sequential nonlinear feature extraction computations enhance relevant cues while removing non-informative sources of noise, thus enhancing the representation of sounds in noise impoverished conditions.

## Author summary

The brain’s ability to recognize sounds in the presence of competing sounds or background noise is essential for everyday hearing tasks. How the brain accomplishes noise resiliency, however, is poorly understood. Using neural recordings from the ascending auditory pathway and an auditory spiking network model trained for sound recognition in noise we explore the computational strategies that enable noise robustness. Our results suggest that the hierarchical feature organization of the ascending auditory pathway and the resulting computations are critical for sound recognition in the presence of noise.

## Introduction

Being able to identify sounds in the presence of background noise is essential for every-day audition and vital for survival. Although peripheral and central mechanisms have been proposed to facilitate robust coding of sounds [1–4] it is presently unclear how the sequential organization of the ascending auditory pathway and its sequential nonlinear transformations contribute to sound recognition in the presence of background noise.

Several hierarchical changes in spectral and temporal selectivity are consistently observed in the ascending auditory pathway of mammals. Temporal selectivity and resolution change dramatically over more than an order of magnitude, from a high-resolution representation in the cochlea, where auditory nerve fibers synchronize to temporal features of up to ~1000 Hz, to progressively slower (limited to ~25 Hz) and coarser resolution representation as observed in auditory cortex [5]. Furthermore, although changes in spectral selectivity can be described across different stages of the auditory pathway, and spectral resolution is somewhat coarser in central levels, changes in frequency resolution are somewhat more homogeneous and less dramatic [6–8]. It is plausible that such hierarchical transforms across auditory nuclei are essential for feature extraction and ultimately high-level auditory tasks such as acoustic object recognition.

We report that the hierarchical organization of the auditory pathway and several sequential nonlinear feature extraction computations are predicted by a spiking auditory network model of the auditory pathway trained to identify speech words for multiple talkers in background noise. The sequential changes in spectro-temporal selectivity and nonlinear transformations of the network mirror those seen in neural data across sequentially organized auditory nuclei (auditory nerve, midbrain, thalamus and cortex). Finally, comparisons of the optimal auditory network with a model designed to maximize information transfer and conventional receptive field based models demonstrates that the sequential transformations of the optimal network enhance sound recognition performance in the presence of competing background noise.

## Results

### Task optimized hierarchical spiking neural network predicts auditory system organization

**Fig 1:**
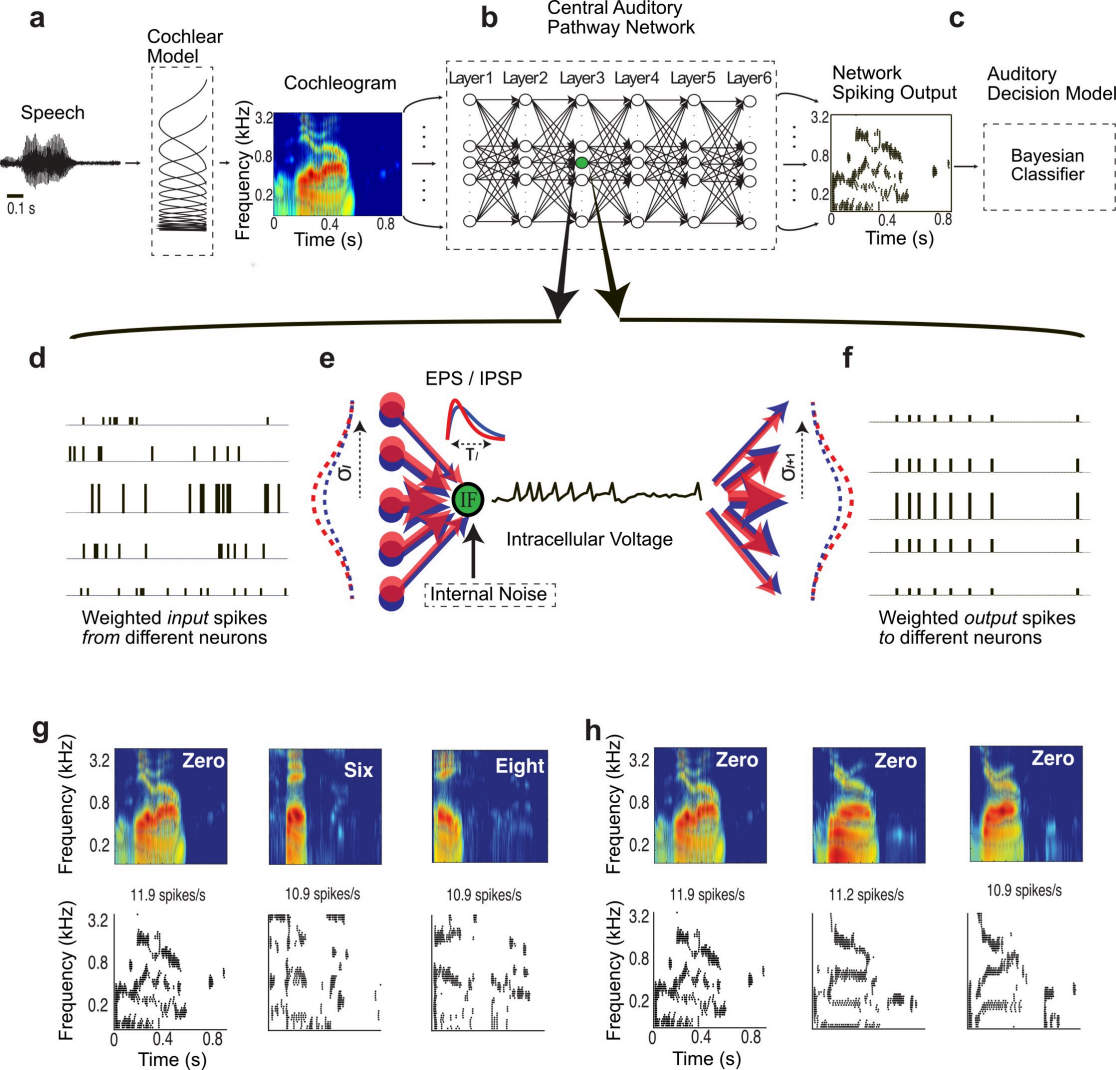
Auditory *hierarchical spiking neural network* (HSNN) model. The model consists of a (**a**) cochlear model stage that transforms the sound waveform into a spectrogram (time vs. frequency), (**b)** a central hierarchical spiking neural network containing frequency organized spiking neurons and a (**c**) Bayesian classifier that is used to read the spatio-temporal spike train outputs of the HSNN. Each dot in the output represents a single spike at a particular time-frequency bin. (**d**-**f**) Zoomed in view of the HSNN illustrates the pattern of convergent and divergent connections between network layers for a single leaky integrate-and-fire (LIF) neuron. (**d-e**) Input spike trains from the preceding network layer are integrated with excitatory (red) and inhibitory (blue) connectivity weights that are spatially localized and model by Gaussian functions (**f**). The divergence and convergence between consecutive layers is controlled by the connectivity width (SD of the Gaussian model, *σ*_*l*_). Each incoming spike generates excitatory and inhibitory post-synaptic potentials (EPSP and IPSP, red and blue kernels in **e**). The integration time constant (*τ*_*l*_) of the EPSP and IPSP kernels can be adjusted to control the temporal integration between consecutive network layers while the spike threshold level (*N*_*l*_) is independently adjusted to control the output firing rates and the overall neuron layer sensitivity. (**g**, **h**) Example cochlear model outputs and the corresponding multi-neuron spike train outputs of the HSNN under the influence of speech babble noise (at 20 dB SNR). (**g**) HSNN response pattern for one sample of the words *zero*, *six*, and *eight* illustrate output pattern variability that can be used to differentiate words. (**h**) Example response variability for the word *zero* from multiple talkers in the presence of speech babble noise (20 dB SNR).

We developed a physiologically motivated hierarchical spiking neural network (HSNN) and trained it on a behaviorally relevant word recognition task in the presence of background noise and multiple talkers. Like the auditory pathway, the HSNN receives frequency-organized input from a cochlear stage (Fig 1**A**) and maintains its topographic (tonotopic) organization through a network of frequency organized integrate-and-fire spiking neurons (Fig 1**B**). For each sound, such as the word “zero”, the network produces a dynamic spatio-temporal pattern of spiking activity (Fig 1**B**, right) as observed for peripheral and central auditory structures [9–11]. Each neuron is highly interconnected containing frequency specific and co-tuned excitatory and inhibitory connections [12–15] that project across six network layers (Fig 1**B**). Converging spikes from neurons in a given layer (Fig 1**D**) are weighted by frequency localized excitatory and inhibitory connectivity functions and the resulting excitatory and inhibitory post-synaptic potentials are integrated by the recipient neuron (Fig 1**D** and 1**E**, note the variable spike amplitudes). Output spike trains from each neuron are then weighted by connectivity function, providing the excitatory and inhibitory inputs to the next layer (Fig 1**E and** 1**F**). The overall multi-neuron spiking output of the network (Fig 1**B**, right; example spiking outputs also shown in Fig 1**G** and 1**H**, bottom) is then treated as a response feature vector and fed to a Bayesian classifier in order to identify the original sound delivered (Fig 1**C**; see Methods).

Given that key elements of speech such as formants and phonemes have unique spectral and temporal composition that are critical for word identification [16,17], we first test how the spectro-temporal resolution and sensitivity of each network layer contribute to word recognition performance in background noise. We optimize the HSNN to maximize word recognition accuracy in the presence of noise and to identify the network organization of three key parameters that separately control the temporal and spectral resolution and the overall sensitivity of each network layer (*l* = 1 … 6). The neuron time-constant (*τ*_*l*_), controls the temporal dynamics of each neuron element in layer *l* and the resulting temporal resolution of the output spiking patterns. The connectivity width (*σ*_*l*_) controls the convergence and divergence of synaptic connections between consecutive layers and therefore affects the spectral resolution of each layer. Since synaptic connections in the auditory system are frequency specific and localized [15,18,19] connectivity profiles between consecutive layers are modeled by a Gaussian profile of unknown connectivity width parameter [20] (Fig 1**E**; specified by the SD, *σ*_*l*_). Finally, the sensitivity and firing rates of each layer are controlled by adjusting the spike threshold level (*N*_*l*_) of each IF neuron [21]. This parameter controls the firing pattern from a high firing rate dense code as proposed for the auditory periphery to a sparse code as has been proposed for auditory cortex [2,22]. Because temporal and spectral selectivities vary systematically and gradually across auditory nuclei [5,8,23], we required that the network parameters vary hierarchically and smoothly from layer-to-layer according to (see Methods: Network Constraints and Optimization)

$$\tau_{l}=\tau_{1}{∙\alpha_{\tau }}^{l-1}$$ (Eq 1)

$$\sigma_{l}=\sigma_{1}{∙\gamma_{\sigma }}^{l-1}$$

$$N_{l}=N_{1}{∙\lambda_{N}}^{l-1}$$

where *τ*_1_, *σ*_1_, and *N*_1_ are the parameters of the first network layer. The first layer parameters are selected to mimic physiologic properties of auditory nerve fibers which have firing rates in excess of several hundred spikes/sec for speech sounds [24], fast temporal processing with short time constants in the order of ~500 *μ*s [25] and narrow tuning bandwidths [6,26] (see Methods). The scaling parameters *α*_*τ*_, *γ*_*σ*_, and *λ*_*N*_ determine the direction and magnitude of layer-to-layer changes for each of the three neuron parameters. A scaling value greater than one indicate that the corresponding neuron parameter increases systematically across layers, a value of one indicates that the parameter is constant forming a network with homogeneous layers, while a value less than one indicates that the parameter value decreases systematically across layers. For instance, a time-constant scaling exponent of *α*_*τ*_ = 2 indicates that the time constants would double from layer-to-layer while a value of *α*_*τ*_ = 0.5 indicates that the time constants is reduced by a factor of 2 from layer-to-layer.

To determine the network architecture required for optimal word recognition in noise and to identify whether such a configuration is essential for noise robust performance, we searched for the network scaling parameters (*α*_*τ*_, *γ*_*σ*_, and *λ*_*N*_) that maximize the network’s word recognition accuracy in a ten-alternative forced choice task (i.e., digit identification) for multiple talkers (eight talkers) and in the presence of speech babble noise (signal-to-noise ratios, SNR = -5, 0, 5, 10, 15, 20 dB; see Methods). Sounds in the optimization and validation corpus consist of spoken words for digits from zero to nine from eight talkers (TI46 LDC Corpus [27], see Methods). For each input sound, the network spike train outputs are treated as response feature vectors and a Bayesian classifier (Fig 1C; see Methods) is used to read the network outputs and report the identified digit (*zero* to *nine*). The Bayesian classifier treats the responses from each neuron at each time bin as independent observations (1 or 0, representing spike or no spike). The probability that the network response belongs to a particular sound (the *posterior*) is then obtained by accumulating the response probabilities over all neurons and time bins, and the sound with maximum posterior is the selected digit (see Methods).

The optimal network outputs preserve important time-frequency information in speech despite variability in the input sound. Example cochlear model spectrograms and the network spiking outputs are shown in Fig 1**G** and 1**H** for the words *zero*, *six*, and *eight* in the presence of speech babble noise (optimal outputs at SNR = 20 dB). Analogous to auditory cortex responses for speech [9], the network produces a distinguishable spiking output for each sound that is frequency specific and reflects its spectro-temporal composition (Fig 1**G**). When a single word is generated by different talkers in noise (SNR = 20 dB) the network produces a relatively consistent frequency organized firing pattern (Fig 1**H**) such that the response timing and active neuron channels remain relatively consistent. As expected, a lack of activity is observed for neurons between ~1–4 kHz within the first ~100–200 ms of the sound for the word *zero* and several time-varying response peaks indicative of the vowel formants are observed for all three talkers (Fig 1**H**).

**Fig 2:**
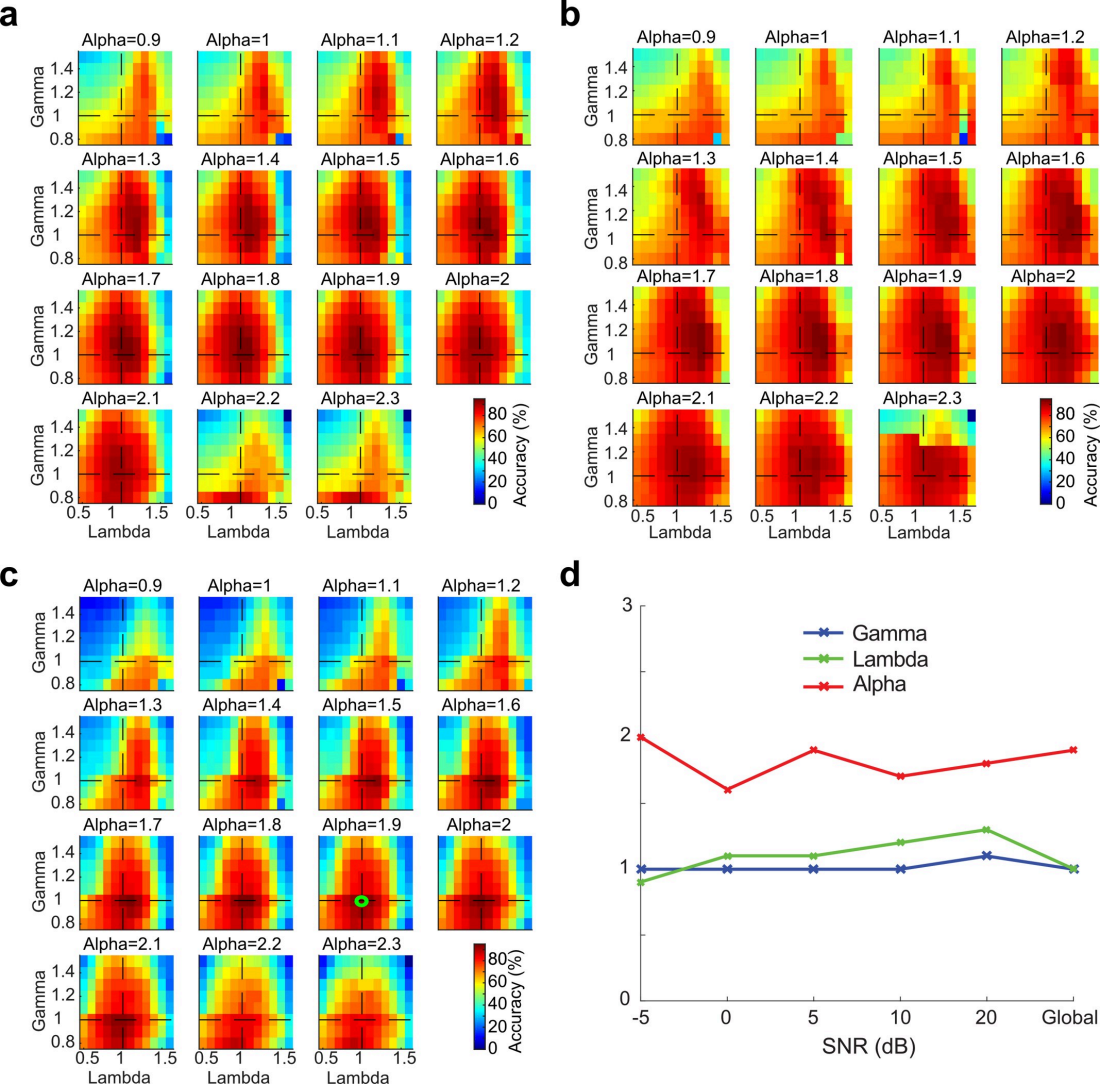
Global optimal solution that maximizes word recognition accuracy in the presence of background noise (-5, 0, 5, 10, 15 and 20 dB SNR). Cross-validated word recognition accuracy (see Methods) is measured using the network outputs as a function of the three scaling parameters (*α*_*τ*_,*γ*_*σ*_, and *λ*_*N*_). Word recognition accuracy curves are shown at 5 and 20 dB SNR (**a** and **b**, respectively) as well as for the global solution (**c**, average accuracy between -5 and 20 dB SNR). In all cases shown, word recognition accuracy curves are tuned for the different scaling parameters and exhibit a similar optimal solution (green circles). (**d**) The optimal scaling parameters are relatively stable across SNRs and similar to the solution that maximize average performance across all SNRs (optimal solution *α*_*τ*_ = 1.9, *γ*_*σ*_ = 1.0, and *λ*_*N*_ = 1.0).

The network word recognition accuracy is shown in Fig 2 as a function of each of the network parameters (*α*_*τ*_, *γ*_*σ*_, and *λ*_*N*_) and SNR (**a**, SNR = 5 dB; **b**, SNR = 20 dB; **c**, average accuracy across all SNRs). At each SNR the word recognition accuracy profiles are tuned with the scaling parameter (i.e., concave function) which enables us to find an optimal scaling parameters that maximizes the classifier performance. Regardless of the SNR the optimal HSNN parameters are relatively constant (Fig 2**D**; tested between -5 to 20 dB) implying that the network organization is relatively stable and invariant of the SNR (Fig 2**A**–2**C**; **a =** 5 dB SNR, **b** = 20 dB SNR, **c** = average across all SNRs). Intriguingly, several functional characteristics of the optimal network mirror changes observed in the auditory pathway. Like the ascending auditory pathway where synaptic potential time-constants vary from sub-millisecond in the auditory nerve to tens of milliseconds in cortex [15,25,28,29], time constants scale in the optimal HSNN (global optimal *α*_*τ*_ = 1.9) over more than an order of magnitude between the first and last layers (1.9^5^ = 24.8 fold increase between the first and last layer; ~0.5 to 12.5 ms) indicating that temporal resolution becomes progressively coarser in the deep network layers. By comparison, the optimal connectivity widths do not change across layers (*γ*_*σ*_ = 1.0). This result suggests that for the optimal HSNN temporal resolution changes dramatically while spectral resolution remains relatively constant across network layers, mirroring changes in spectral and temporal selectivity observed along the ascending auditory pathway [5–8].

**Fig 3:**
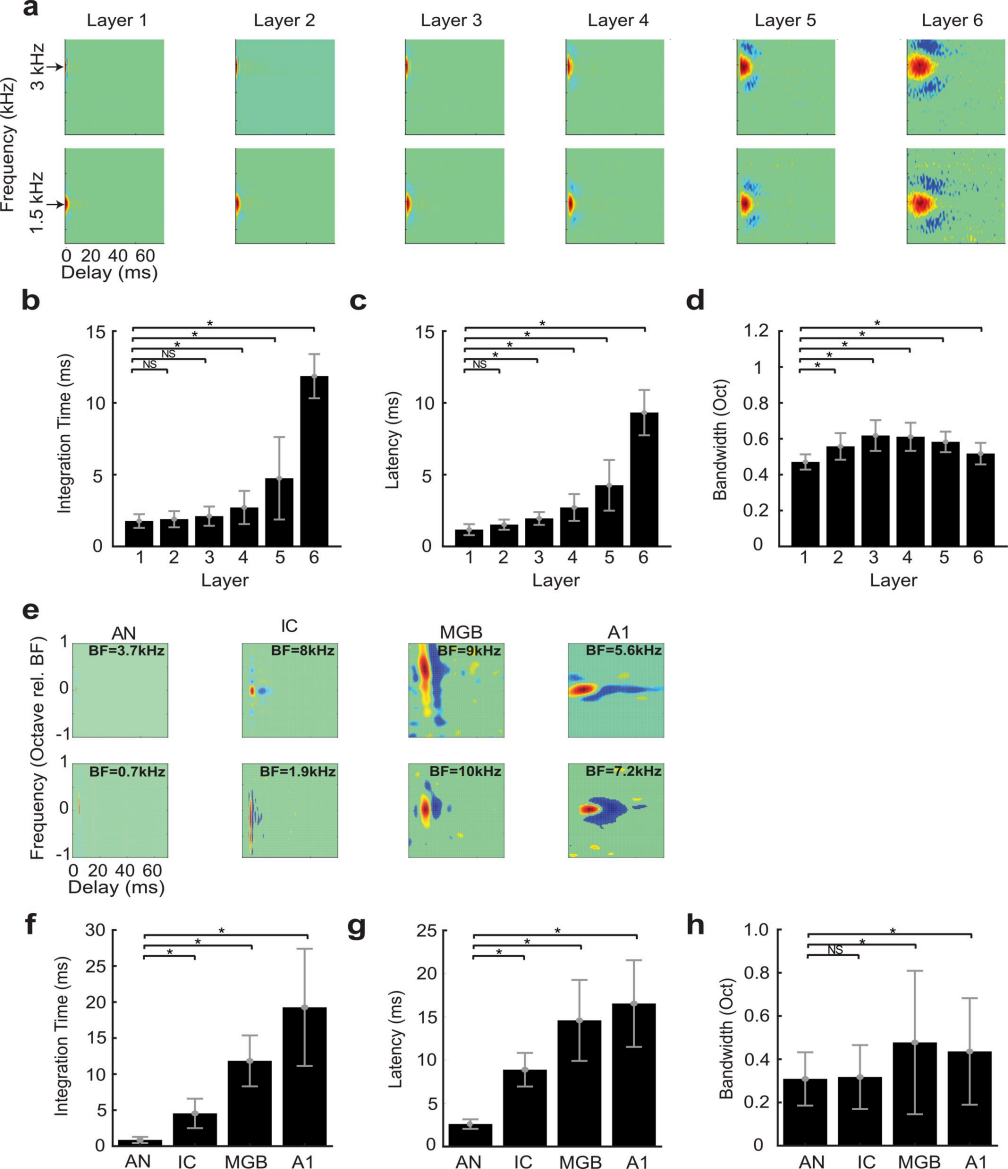
Receptive field transformations of the optimal HSNN predicts transformations observed along the ascending auditory pathway. (**a**) Example spectro-temporal receptive field (STRF) measured for the optimal network change systematically between consecutive network layers. All STRFs are normalized to the same color scale (red = increase in activity or excitation; blue = decrease in activity or inhibition/suppression; green tones = lack of activity). In the early network layers STRFs are relatively fast with short duration and latencies, and relatively narrowly tuned. STRFs become progressively slower, slightly broader, and have longer patterns of inhibition across the network layers, mirroring changes in spectral and temporal selectivity observed in the ascending auditory pathway. The measured (**b**) integration times, (**c**) latencies, and (**d**) bandwidths increase across the six network layers. (**e**) Examples STRFs from the auditory nerve (AN) [26], inferior colliculus (IC) [7], thalamus (MGB) and primary auditory cortex (A1) [8] become progressively longer and have progressively more complex spectro-temporal sensitivity along the ascending auditory pathway. Average integration times (**f**), latencies (**g**) and bandwidths (**h**) between AN and A1 follow similar trends as the optimal HSNN (**b**-**d**). Asterisks (*) designate significant comparisons (t-test with Bonferroni correction, p<0.01) relative to layer 1 for the optimal network (**b**-**d**) or relative to the auditory nerve for the neural data (**f**-**h**) while error bars designate SD.

The scaling parameters of the optimal HSNN indicate a substantial loss of temporal (*α*_*τ*_ = 1.9) and no change in connectivity resolution (*γ*_*σ*_ = 1.0) across network layers. This prompted us to ask how feature selectivity changes across the network layers and whether a sequential transformation in spectral and temporal selectivity improve word recognition performance in noise. To quantify the sequential transformations in acoustic processing, we first measure the spectro-temporal receptive fields (STRFs) of each neuron in the network (see Methods). Example STRFs are shown for two selected frequencies across the six network layers (Fig 3**A**; best frequency = 1.5 and 3 kHz). As a comparison, example STRFs from the auditory nerve (AN) [26], midbrain (inferior colliculus, IC) [7], thalamus (MGB) and primary auditory cortex (A1) [8] of cats are shown in Fig 3**E**. Like auditory pathway neurons, STRFs from the optimal HSNN contain excitatory domains (red) with temporally lagged and surround inhibition/suppression (blue) along the frequency dimension (Fig 3**A**). Furthermore, STRF varied substantially across layers (integration time, latency, bandwidth; MANOVA, F(5,131) = 65, p<1x10^-10^) being substantially faster in the early network layers lasting only a few milliseconds and mirroring STRFs from the auditory nerve, which have relatively short latencies and integration times. STRFs have progressively longer integration times (paired t-test with Bonferroni correction, p<0.01; Fig 3**B**) and latencies (paired t-test with Bonferroni correction, p<0.01; Fig 3**C**) across network layers, while bandwidths vary only slightly from the first to last layer (paired t-test with Bonferroni correction, p<0.01; Fig 3**D**). These sequential transformations mirror changes in temporal and spectral selectivity seen between the auditory nerve, midbrain, thalamus and ultimately auditory cortex (Fig 3**E**–3**H**). As for the auditory network model, integration times (Fig 3**F**) and latencies (Fig 3**G**) increase systematically and smoothly (paired t-test with Bonferroni correction, p<0.01) while bandwidths show a small but significant increase between the auditory nerve and cortex (paired t-test with Bonferroni correction, p<0.01), analogous to results from the computational network.

### Hierarchical and nonlinear transformations enhance recognition in noise

**Fig 4:**
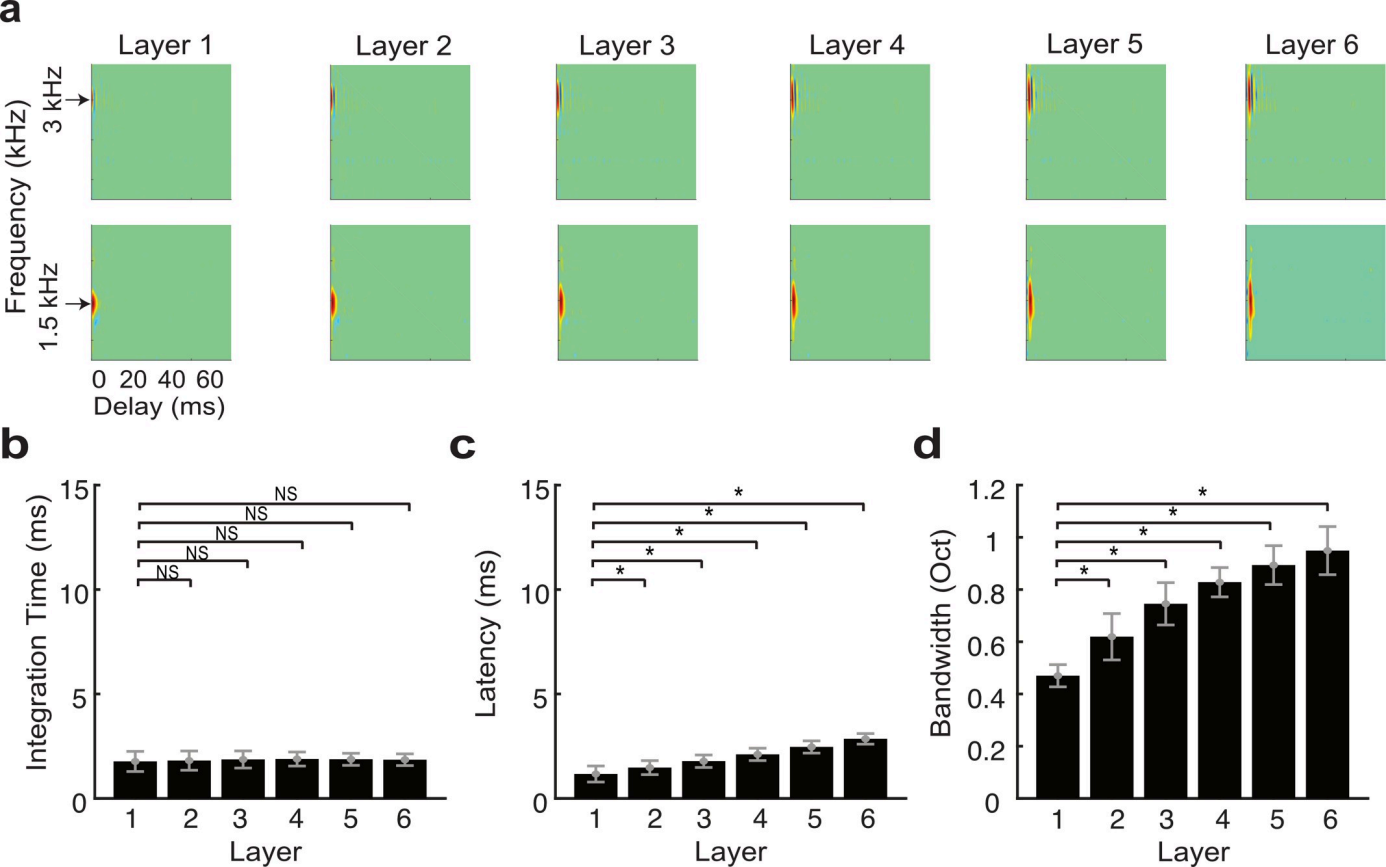
Receptive field transformations of the high-resolution network indicate that spectro-temporal information propagates with minimal processing across network layers. (**a**) Example spectro-temporal receptive field (STRF) measured for the optimal network maintain high-resolution and change minimally across network layers. Unlike the optimal network, the measured (**b**) integration times and (**c**) latencies change minimally and are relatively constant across the six network layers. (**d**) Bandwidths, by comparison, increase slightly across the six network layers. The figure format follows the same convention as in Fig 3.

It is intriguing that the dramatic reduction in temporal and subtle reduction in spectral resolution in the optimal network mirror changes in selectivity observed in the ascending auditory system, as this ought to limit the transfer of acoustic information across the network. In particular, there is no *a priori* reason for the network integration parameters to scale in the temporal domain over nearly two orders of magnitude between the first and last layer (*α*_*τ*_ = 1.9) whereas similar scaling is not observed in the spectral domain (*γ*_*σ*_ = 1), thus producing changes in spectrotemporal selectivity observed physiologically. One plausible hypothesis is that the sequential changes in spectro-temporal selectivity created by the network organization are necessary to extract invariant acoustic features in speech, such as formants and consonant-vowel transitions that are needed for word identification, while rejecting noise and fine details in the acoustic signal that may contribute in a variety of hearing tasks (e.g., spatial hearing, pitch perception, etc.), but ultimately don’t contribute to speech recognition performance. This may be expected since human listeners require a limited set of temporal and spectral cues for speech recognition [16,17] and can achieve high recognition performance even when spectral and temporal resolution is degraded [30,31]. We thus tested the above hypothesis by comparing the optimal network performance against a high-resolution network that lacks scaling (*α*_*τ*_ = 1, *γ*_*σ*_ = 1 and *λ*_*N*_ = 1) and for which we expect a minimal loss of acoustic information across layers. Although the receptive fields of the optimal network change significantly across layers (MANOVA, F(5,131) = 63, p<1x10^-10^), the temporal receptive field parameters of the high-resolution network are relative consistent and change minimally across layers (Fig 4). Integration times do not vary across layers (paired t-test with Bonferroni correction, NS) whereas latencies increase only slightly between layer 1 and 6 (paired t-test with Bonferroni correction, p<0.01). Furthermore, despite the fact that network connectivity widths are constant across layers, STRF bandwidths increase systematically (paired t-test with Bonferroni correction, p<0.01). Together, this suggests that temporal information propagates across the high-resolution network with minimal processing while spectral information becomes substantially degraded across layers.

**Fig 5:**
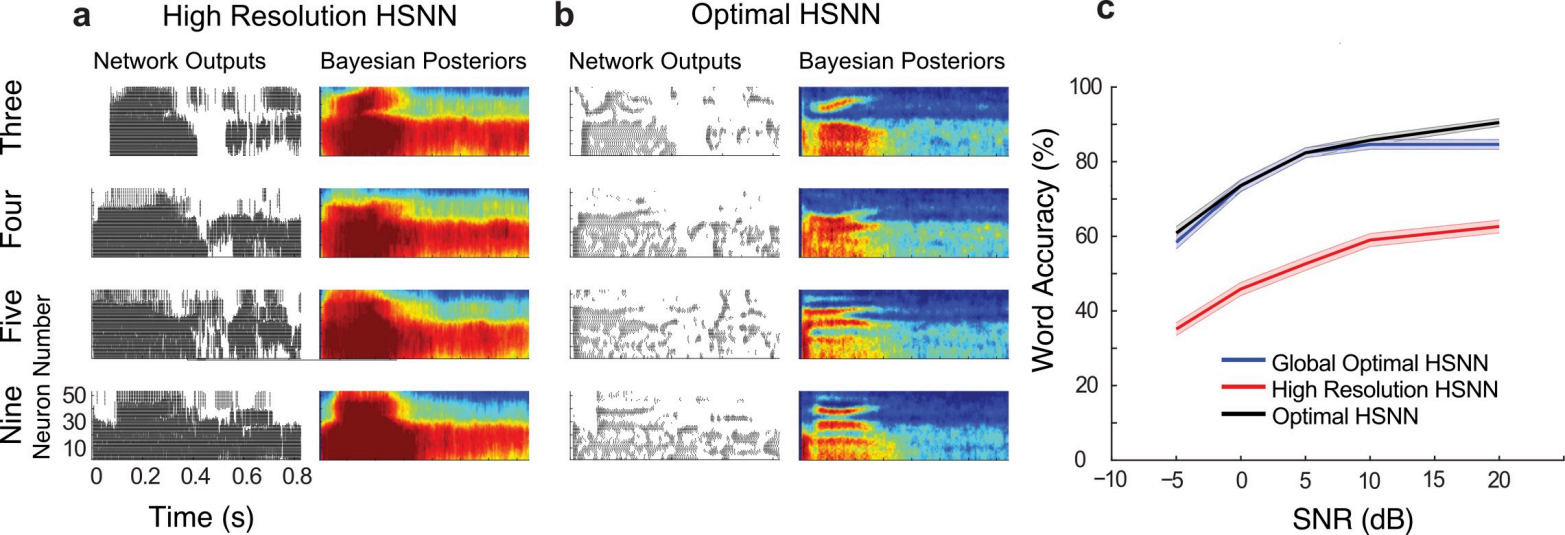
Optimal HSNN outperforms a high-resolution HSNN designed to preserve incoming acoustic information. Sample network spike train outputs and Bayesian likelihood histograms for the words *three*, *four*, *five*, and *nine* are shown for the (**a**) high-resolution and (**b**) optimal HSNN at 5 dB SNR. The Bayesian likelihood histograms correspond to the average probability of firing at each time-frequency bin for each digit (averaged across all trials and talkers). The firing patterns and Bayesian likelihood of the high-resolution network are spatio-temporally blurred compared to the hierarchical network. (**b**) Details such as spectral resonances (e.g., formants) and temporal transitions resulting from voicing onset are accentuated in the hierarchical network output. (**c**) The optimal HSNN (maximize performance across all SNRs) outperforms the high-resolution network in the word recognition task at all SNRs tested (blue = optimal; red = high-resolution) with an average accuracy improvement of 25.7%. The optimal HSNN word recognition accuracy also closely matches the performance when the network is optimized and tested individually at each SNR (black, SNR optimal HSNN) indicative of a stable network representation.

Fig 5 illustrates how the optimal HSNN accentuates critical spectral and temporal cues necessary for speech recognition while the high-resolution network fails to do the same. Example Bayesian likelihood time-frequency histograms (average firing probability across all excerpts of each sound at each time-frequency bin) measured at 5 dB SNR are shown for the words “three”, “four”, “five” and “nine” for both the high-resolution (Fig 5**A**) and optimal (Fig 5**B**) HSNN along with selected spiking outputs from a single talker. Intriguingly and despite the fact that the high-resolution HSNN ought to preserve fine acoustic information, the Bayesian likelihood for the high-resolution network are highly blurred in both the temporal and spectral dimensions and have similar structure for the example words (Fig 5**A**, right panels). This is also seen in the individual network outputs where the high-resolution network produces a dense and saturated firing pattern (Fig 5**A)** that lacks the detailed spatio-temporal pattern seen in the optimal HSNN (Fig 5**B)**. In sharp contrast, the optimal HSNN preserves and even accentuates key acoustic elements such as temporal transitions for voice onset timing and spectral resonances (formants) while simultaneously rejecting and filtering out the background noise (Fig 5**B**, right panels), ultimately leading to enhanced word recognition accuracy in noise (Fig 5C).

**Fig 6:**
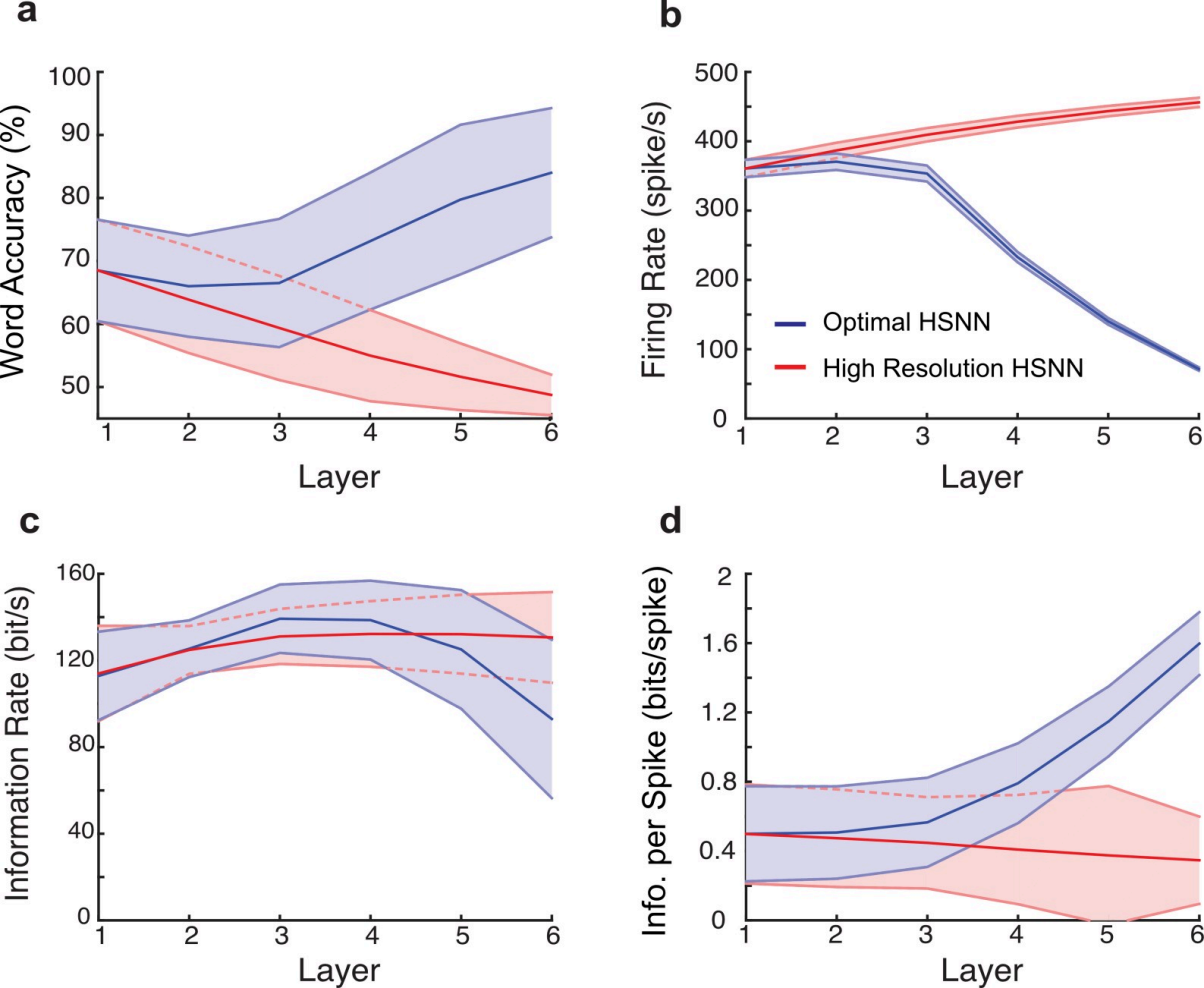
Hierarchical transformation between consecutive network layers enhances word recognition performance and robustness of the optimal HSNN. (**a**) The average word accuracy at 5 dB SNR systematically increases across network layers for the optimal HSNN (**a**, blue) whereas the high-resolution HSNN exhibits a systematic reduction in word recognition accuracy (**a**, red). For the high-resolution HSNN average firing rates (**b**, red), information rates (**c**, red), and information per spike (**d**, red) are relatively constant across layers indicating minimal transformations of the incoming acoustic information. In contrast, average firing rates (**b**, blue) and information rates (**c**, blue) both decrease between the first and last network layers of the optimal network, consistent with a sequential sparsification of the response and a reduction in the acoustic information encoded in the output spike trains. However, the information conveyed by single action potentials (**d**, blue) in the optimal HSNN sequentially increase between the first and last layer so that individual action potentials become progressively more informative across layers. Continuous curves show the mean whereas error contours designate the SD.

To characterize the transformations of the HSNN that enhance word recognition accuracy, we examine how acoustic information propagates and is transformed across sequential network layers. For each layer, the spike train outputs are first fed to the Bayesian classifier in order to measure sequential changes in word recognition accuracy. In the optimal HSNN, word recognition accuracy systematically increases across layers with an average improvement of 15.5% between the first and last layer when tested at 5 dB SNR (p<0.001, t-test; Fig 6**A**, blue; 13.7% average improvement across all SNRs). By comparison, for the high-resolution HSNN, performance degrades sequentially across layers with an average decrease of 19.8% between the first and last layer (p<0.001, t-test; Fig 6**A**, red; 18.1% average reduction across all SNRs). This suggest that the optimal network architecture is able to preserve and take advantage of critical acoustic information for the word recognition task.

Although the classifier performance takes advantage of the hierarchical organization in the optimal HSNN to selectively enhance word accuracy across layers (Fig 6A), a similar trend is not observed for the transfer of total acoustic information. First, firing rates decrease systematically across layers for the optimal HSNN, consistent with a sparser output representation (Fig 6**B**, blue) as proposed for deep layers of the auditory pathway [2,22,32]. By comparison, firing rates are relatively stable across layers for the high-resolution network (Fig 6**B**, red). We next measure the average mutual information (see Methods) in the presence of noise (5 dB) to identify how incoming acoustic information is sequentially transformed from layer-to-layer. For the optimal HSNN the information rates (i.e., bits / sec) decreases between the first and last layer (Fig 6**C**, blue) whereas for the high-resolution network information is conserved across network layers (Fig 6**C**, red). Thus, the layer-to-layer increase in word recognition accuracy observed for the optimal HSNN is accompanied by a loss of total acoustic information in the deep network layers. However, although total information decreases across layers, the information conveyed by single action potentials is higher and increases across layers (Fig 6**D**, blue). This contrast the high-resolution HSNN where information per spike remains relatively constant across layers (Fig 6**D**, red). This suggests that individual action potentials become increasingly more informative from layer-to-layer in the optimal HSNN despite a reduction in firing rates and total information. Taken together with the changes in spectro-temporal selectivity (Fig 3), the findings are consistent with the hypothesis that the optimal HSNN produces a reliable sparse code in which invariant acoustic features are represented with isolated spikes. By comparison, the high-resolution network produces a dense response pattern that has a tendency to preserve incoming acoustic information, including the background noise and nonessential acoustic features, thus suffering in recognition performance.

**Fig 7:**
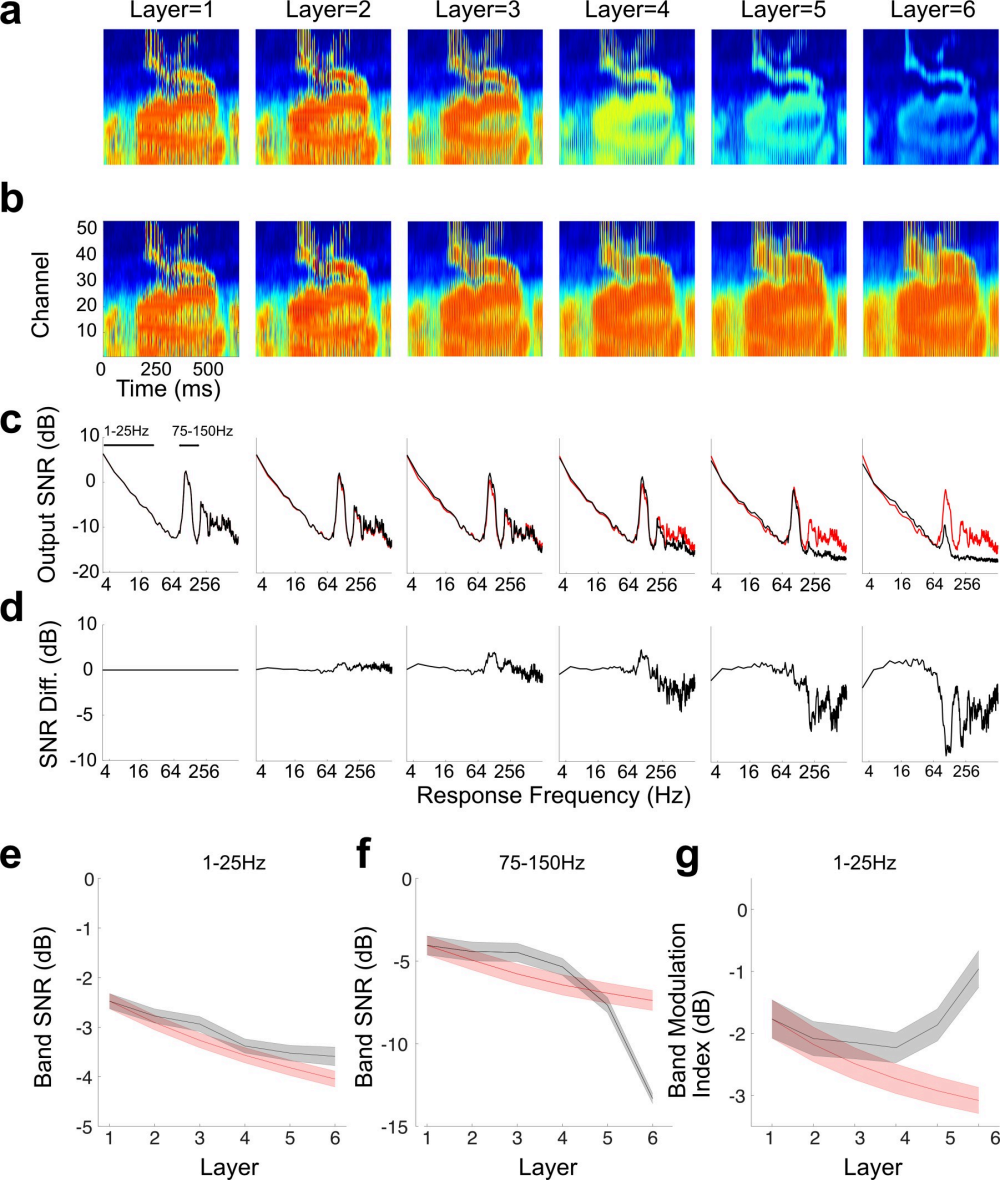
Hierarchical transformation between consecutive network layers of the optimal HSNN serve to denoise the speech signal and selectively enhance word related temporal information. The average network outputs are shown for the word zero (over 50 trials) at different layers of the optimal (**a**) and high-resolution networks (**b**). The response signal-to-noise ratio (SNR) remains relatively consistent for the high-resolution network across layers (**c**, red curve**)**. By comparison, for the optimal network the response is sequentially lowpass filtered so that the response SNR is sequentially reduced across layers for high modulation frequencies (**c**, black curve). (**d**) The difference SNR (optimal–high res, black–red curve in panels **b**) demonstrate a sequential lowpass filtering that accumulates across layers for the optimal HSNN. The band SNR within the fluctuation/rhythm range (1–25 Hz) decreases with layer, but is slightly enhanced for the optimal network (**e**, black curve) when compared to the high-resolution network (**e**, red curve). The band SNR within the periodicity pitch range (75–150 Hz) is substantially reduced across layers of the optimal network (**f**, black) when compared to the high-resolution network (**f**, red). The modulation index within the 1–25 Hz band increases and is thus enhanced across layers of the optimal network (**g**, black) whereas it is reduced for the high-resolution network (**g**, red). Error bars in panels **e**-**g** represent SEM.

We next explored the sequential changes in the feature representation of the optimal and high resolution networks. Fig 7**A** and 7**B** shows the average outputs of the optimal and high-resolution networks, respectively, for an utterance of the word zero at 5dB SNR from one talker (averaged over 50 simulation trials using different segments of babble noise for each trial). Several structural differences can be seen visually between the two networks. First, although the response strength is relatively consistent for the high-resolution network across layers (**b**) it becomes progressively weaker starting with layer four of the optimal network (**a**, consistent with a sparser output). Second, in the early layers, the average response of both networks exhibit vertical bands of synchronous activity. These temporally fast and synchronized responses reflect the vocal fold periodicity of the stimulus, which falls in the range of ~100 Hz for the male talker analyzed. For the high-resolution network, this periodicity response is largely preserved across layers; however, for the optimal network this periodicity response degrades and is largely abolished by the six layer. Finally, a relatively strong sustained background of activity, which reflects responses to the background noise, is observed in the early network layers, particularly for channels 1–25 of the networks. Although this sustained activity is largely preserved for the high-resolution network across layers, it is progressively weakened and is largely removed by layer six of the optimal HSNN.

The structural changes observed visually for the word zero were reflected in the measured response signal-to-noise ratio (SNR), which varies systematically for the optimal network across layers but remains relatively consistent for the high-resolution network (Fig 7**C**; see Methods). In the first network layer, the SNR contains two dominant peaks reflecting critical perceptual ranges for temporal envelope in speech [5,31,33](Fig 7**C**). The fluctuation/rhythm range (analyzed between 1–25 Hz) encompasses slow temporal fluctuations in speech such as those created by words, phonemes, and syllables and which is critical for word recognition. The periodicity pitch range (analyzed for 75–150 Hz) encompasses substantially faster envelope fluctuations created by vocal fold vibration that are known to contribute to voice quality. Although the SNR curves remain relatively consistent across layers for the high-resolution network regardless of response frequency, synchronization to high frequencies systematically degrades across layers of the optimal network, and much of the periodicity pitch range signal is attenuated at layer six. The difference SNR curve (optimal–high-res; Fig 7**D**) demonstrates that both networks similarly preserve temporal information out to ~60 Hz (~ 0 dB difference). However, the optimal network progressively lowpass filters fast temporal envelopes beyond ~60 Hz. These systematic changes in the representation of the slow and fast envelopes were quantified by measuring the band averaged SNR for the fluctuation (1–25 Hz; Fig 7**E**) and periodicity pitch (75–150 Hz; Fig 7**F**) ranges. The fluctuations band SNR (1–25 Hz) follows a similar trend across layers for both networks, although it is slightly higher for optimal HSNN (0.5 dB; Fig 7**E**; MANOVA, F(1,104) = 24.7, p<0.001). By comparison, there was substantially larger reduction of 6 dB in the band SNR within the periodicity range (75–150 Hz) for the optimal network (Fig 7**F**; MANOVA, F(1,104) = 285, p<1x10^-57^) indicating that information related to the fast temporal envelopes is sequentially removed by the optimal HSNN. Finally, the response band modulation index (see Methods), which captures the temporal variation of the output relative to the sustained response (DC component), was significantly enhanced by 2.1 dB across layers for the optimal network when compared to the high resolution HSNN (Fig 7**G**; MANOVA, F(1,104) = 187, p<1x10^-37^). Thus, the high-resolution HSNN preserves much of the incoming temporal acoustic information, including fast temporal fluctuations in the periodicity pitch range and background noise (reflected in the DC response), which limits its performance. This contrast the optimal network, which primarily preserves and enhances low modulation frequency information in the fluctuation range while simultaneously discarding temporal details in the periodicity pitch range and background noise.

**Fig 8:**
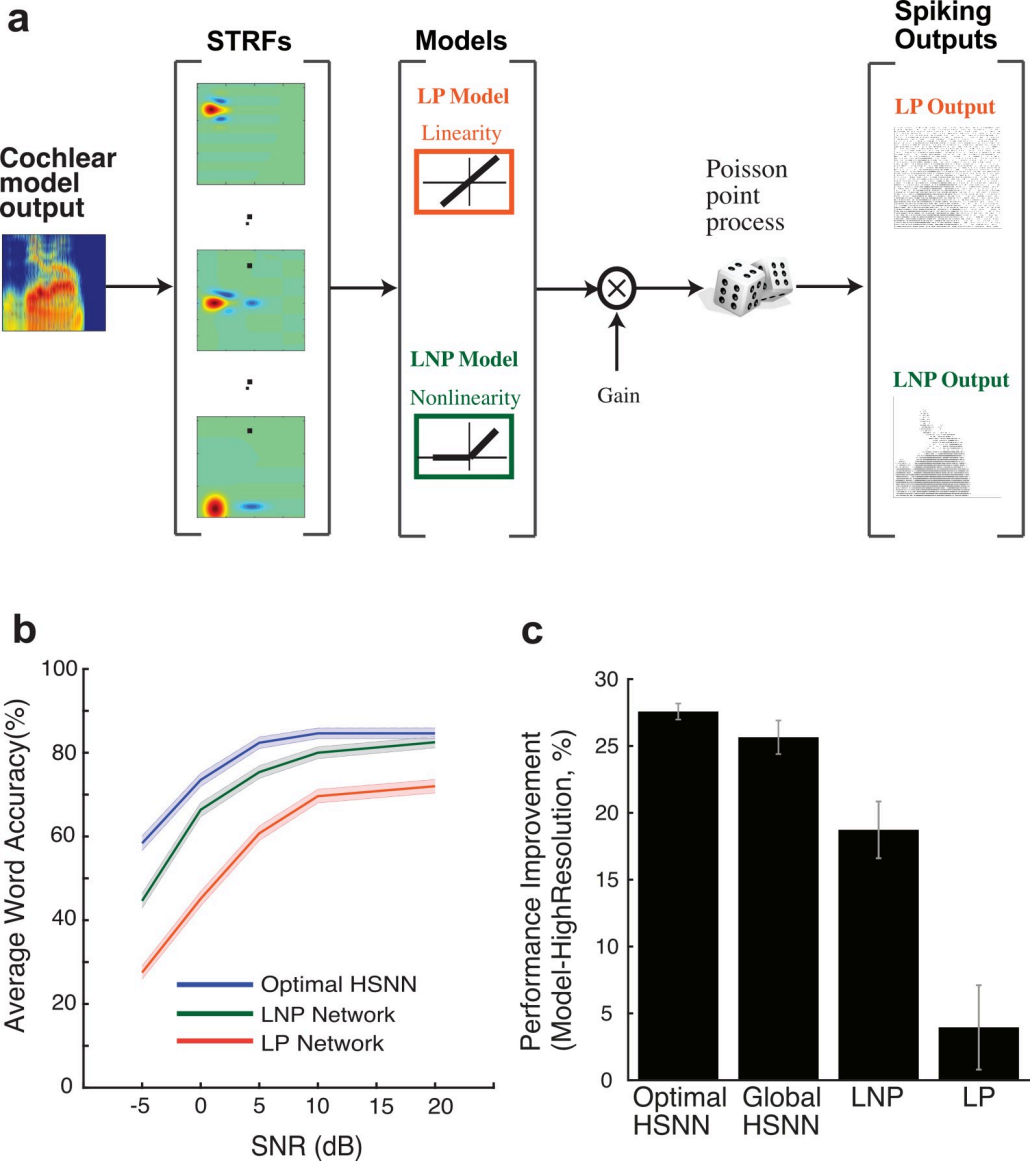
Optimal HSNN enhances robustness and outperforms single-layer generalized linear model networks with matched linear and nonlinear receptive field transformation. (**a**) Linear STRFs obtained at the output of the HSNN are used as to model the linear receptive field transformation of each neuron (see Methods). The LP network consists of an array of linear STRFs followed by a Poisson spike generator. The LNP network additionally incorporates a rectifying output stage following each STRF. (**b**) The optimal HSNN outperformance the LP network with an average performance improvement of 21.7% across SNRs. Nonlinear output rectification in the LNP network improves the performance to within 2% of the HSNN at 20 dB SNR. However, the average LNP performance was 7% lower than the optimal HSNN and performance degraded systematically with increasing noise levels (13.75% performance reduction at -5 dB SNR) demonstrating enhanced robustness of the optimal HSNN. (**c**) Performance improvement of each of the tested models compared against the performance of the high-resolution network.

Given the observed layer-to-layer changes in the optimal HSNN, we next asked whether the sequential layer-to-layer transformations are necessary to enhance word recognition accuracy in noise. Hypothetically, it is plausible that layer-to-layer transformations are not need and similar performance could be achieved with a single layer network as long as each neuron accounts for the overall linear receptive field transformations. To test this, we developed single-layer networks consisting of generalized linear model neurons [34] with either a linear receptive field and Poisson spike train generator (LP network) or a linear receptive field and nonlinear stage followed by Poisson spike train generator (LNP network) (Fig 8**A**; see Methods). The performance of the LP network, which accounts for the linear transformations of the optimal HSNN, was on average 21.7% lower than the optimal HSNN indicating that nonlinearities are critical to achieve high word recognition accuracy (Fig 8**B**). Its plausible that this performance disparity can be overcome by incorporating a nonlinearity that models the rectifying effects in the spike generation process of neurons (LNP network). Doing so improves the performance to within 2.1% of the optimal HSNN when there is little background noise (SNR = 20 dB, 85.6% for optimal HSNN versus 82.5% for LNP network). However, the LNP performance degraded when background noise was added when compared to the optimal HSNN, with an overall performance reduction of 13.8% at -5 dB SNR (58.4% for optimal HSNN versus 44.6% for LNP network). Furthermore, the optimal HSNN outperformed all other models tested across conditions. The LNP, LP, and high-resolution HSNN exhibited a rank order reduction in average performance (Fig 8**C**, 7%, 21.7%, 25.7% respectively; p<0.05, t-test with Bonferroni Correction). Overall, the findings indicate that although the linear and nonlinear receptive field transformations both contribute to the overall network performance, the sequential layer-to-layer transformations carried out by the optimal HSNN improve the performance in the presence of noise.

### Optimal spiking timing resolution

**Fig 9:**
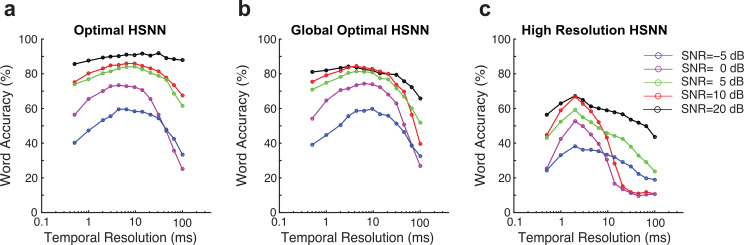
Optimal temporal resolution that maximize word recognition accuracy in noise. (**a**) Word accuracy rate as a function of spike train temporal resolution (bin widths 0.5–100 mms) and SNR (-5 to 20 dB) for the optimal (**a**) and high-resolution networks (**c**). Each curve is computed by selecting the optimal scaling parameters for each SNR and measuring the word accuracy rate from the network outputs at multiple temporal resolutions. (**b**) Same as (**a**), except that global optimal scaling parameters were used for all SNRs tested. The temporal resolution that maximizes the word accuracy rate of the global optimal HSNN is 6.5 ms. (**c**) Word accuracy rate as a function of temporal resolution and SNR for the high-resolution network. The optimal temporal resolution for the high-resolution HSNN is 2 ms.

Finally, we identified the spike timing resolution required to maximize recognition accuracy as previously identified for decoding neural activity in auditory cortex [9,35]. To do so, we synthetically manipulating the temporal resolution of the output spike trains while measuring the word recognition accuracy at multiple SNRs (see Methods). An optimal spike timing resolution is identified within the vicinity of 4–14 ms for the optimal network (Fig 9**A** and 9**B**) which is comparable to spike timing precision required for sound recognition in auditory cortex [9,35]. By comparison, the high-resolution network requires a high temporal resolution of ~2 ms to achieve maximum word accuracy (46.6% accuracy across all SNRs; Fig 9**C**), which is ~ 31.8% lower on average than the optimal network (78.4% accuracy for the optimal HSNN across all SNRs). Taken across all SNRs, the optimal temporal resolution that maximized word accuracy rates is 6.5 ms, which is comparable to the spike timing resolution reported for optimal speech and vocalizations recognition in auditory cortex [9,35].

## Discussion

The results demonstrate that the hierarchical organization of the ascending auditory system is consistent with a feature extraction strategy that enhances sound recognition performance in the presence of noise. Upon optimizing the network organization on a behaviorally relevant word recognition task, the HSNN achieves high recognition accuracy by sequentially refining the spectral and temporal selectivity from layer-to-layer and predicts spectro-temporal transformations observed along the auditory pathway. Comparable word recognition accuracy was not achieved using a spiking network that lacks scaling and conventional receptive field based networks even when the receptive fields capture the linear integration of the optimal HSNN and a threshold nonlinearity was imposed. The sequential nonlinear transformations of the optimal HSNN preserve critical acoustic features for speech recognition while simultaneously discarding acoustic noise and fast modulations not relevant to the sound recognition task. These transformations mirror changes in selectivity along the ascending auditory pathway, including an extensive loss of temporal resolution [5], slight loss of spectral resolution [6–8], and increase in sparsity [2,22]. Thus, the simulations suggest that the orderly arrangement of receptive fields and sequential nonlinear transformations of the ascending auditory pathway improve recognition in noise.

Critical to our findings is the observation that the optimal network transformations described here are not expected a priori as a general sensory processing strategy and may in fact be unique to audition. For instance, changes in temporal selectivity between the retina, visual thalamus, and visual cortex are generally small and neurons in the visual pathway synchronize over a relatively narrow range of frequencies (typically < 20 Hz) [36–39]. This differs dramatically from the nearly two orders of magnitude increase in integration times reported here and the systematic increase in time-constants ranging from microsecond time scales in auditory nerve to tens of milliseconds in auditory cortex [15,25,28,29]. Such a broad range of time-scales is critical in audition, since the physical temporal cues in sounds vary over several orders of magnitude extending from as little as hundredths of μsec for interaural time delays, a few milliseconds for fine structure and periodicities cues, to tens or hundredths of milliseconds for rhythmic fluctuations. By comparison, in the spatial domain, the divergence and convergence of connections in the optimal HSNN is relatively stable producing only a subtle change in receptive field bandwidths between layers, comparable to transformations observed between the auditory nerve and cortex [6–8,23,26]. This contrasts the connectivity of visual system, where there is substantial divergence in connectivity between the retina and visual cortex since visual receptive fields sequentially grow in size between the periphery and cortex so as to occupy a larger area of retinotopic space [40–42].

It is surprising that the optimal HSNN configuration for speech recognition replicates sequential transformations observed along the ascending auditory pathway, given that the auditory pathway is substantially more complex and that the HSNN itself lacks various key auditory pathway transformations such as descending feedback [43] and adaptive nonlinearities [1,3,44]. In particular, it is intriguing that the sequential changes in temporal resolution closely mirror those observed in the auditory pathway. For one, the average integration times between IC (4.5 ms), thalamus (11.8) and auditory cortex (19.2 ms) increase by roughly a factor of two between layers, which is consistent with the optimal time constant scaling exponent of *α*_*τ*_ = 1.9. Furthermore, extrapolating from the auditory nerve average integration time (0.9 ms) to IC where there are anatomically three intermediate auditory structures (cochlear nucleus, superior olive, lateral lemniscus) we would predict a time constant scaling exponent of 1.7 ((4.5/.9)^1/3^) which is comparable to time constant scaling exponent of the optimal network.

Although auditory receptive fields can be more diverse than those of the HSNN, the receptive fields of the optimal HSNN nonetheless contain basic features seen across the auditory pathway including lateral inhibition, temporally lagged inhibition or suppression, and sequentially increasing time-constants along the hierarchy [8,26,45–47]. The HSNN employs several computational principles observed anatomically and physiologically, including the presence of spiking neurons, inhibitory connections, cotuning between excitation and inhibition, and a frequency specific localized circuitry, all of which contribute to its performance. For instance, removing inhibition form auditory cortical receptive field models reduces the ability to detect transient sound elements that are critical for encoding complex natural sounds and reduces sound discrimination performance [48]. In our case, we modified the sequential network structure so that it lacks scaling and also considered reduced single layer GLM networks, both of which exhibited lower performance. This indicates that the network organization itself and its sequential nonlinear transformations can play a critical role.

An intriguing difference between the optimal and high-resolution HSNN is the layer-to-layer sparcification of the response observed for the optimal network which mirrors previously proposed sparcification along the auditory pathway [2,22,32]. Although the intracellular threshold level required for spike generation can serve to sparsify neural activity and enhance the selectivity of auditory neurons [21], the threshold values of both the optimal and high-resolution networks are identical and do not change across network layers (*λ*_N_ = 1), so they do not contribute to this effect. Instead, the layer-to-layer sparcification in the optimal HSNN is driven by the sequentially increasing time constants across layers. Following the generation of an action potential, the cell membrane resets to the resting potential and the voltage needs to build up to the threshold value for spike initiation. Neurons at the deep layers of the optimal HSNN, and likewise for the auditory pathway, have longer time constants which requires a longer buildup time to reach threshold to generate action potentials. This longer buildup time thus reduces the firing in the deep layers producing sparser response. Thus, the optimal HSNN sparcification is largely dependent on the time-constants of each layer. This is consistent with previously described relationship between integration-times and sparcification observed between IC and A1 [49] and thus may be a major factor driving sparse activity across auditory pathway.

The sequential nonlinear transformations of the optimal HSNN are critical for enhancing recognition accuracy, since single layer generalized linear models designed to capture the overall linear transformations of the HSNN did not achieve comparable performance especially for low SNR. This was true even though the GLM receptive fields were derived directly from the HSNN output data and firing rates were matched to that of the HSNN. The GLM achieved identical performance as the HSNN at 20 dB SNR. However, GLM accuracy degraded more rapidly than the HSNN with decreasing SNR indicating that HSNN performance was more stable than the GLM upon adding background noise. It could be argued that this is attributed to the high-dimensionality of the HSNN, however, this is unlikely because both networks actually have comparable dimensionality. The model receptive fields used for the GLM neuron models contain 20 independent parameters that have been shown to be necessary for describing the integration of auditory neurons [45]. Given that there are 53 frequency specific sound channels in the GLM model, the GLM model contains a total of ~1060 parameters (53 neurons x 20 parameters / neuron) which is less than although within the same order of magnitude as the HSNN (600 neurons x 3 parameters = 1800). Also, we note that the HSNN was optimized by adjusting only three parameters, which produces a structured network organization with neuron parameters that are correlated within and across layers, such that the overall dimensionality of the HSNN is actually quite lower.

Recent advances in deep neural networks (DNN) and auditory modeling have made it possible to achieve high-levels of sound recognition performance approaching human performance limits and such networks can predict various features of peripheral and central auditory processing [50–52]. Although these networks differ from the proposed HSNN in terms of the computing elements used, the computations performed and the network structure. One critical distinction is that for conventional DNNs feature extraction and classification are sequentially integrated within a single network, whereas the HSNN was designed strictly for feature extraction while classification is carried out separately with a Bayesian classifier. We choose this approach intentionally, so that the classifier does not perform additional signal processing or high-level feature extraction beyond that of the network that could further improve performance. This allows us to study feature extraction separately from classification. We also intentionally constrained the network structure by defining three optimization parameters that directly control for the functional properties of interest (spectral resolution, temporal resolution, and network sensitivity) and which have direct analogs in the physiology (spread of synaptic connections, cell membrane time constants, and threshold levels). Furthermore, in contrast to conventional DNNs which typically have tens of thousands of optimization parameters lending to their high performance, it is actually surprising that the HSNN can achieve relatively high performance given its few degrees of freedoms (thee optimization parameters). The fact that certain combinations of the three HSNN parameters lead to poor performance, whiles others do not, suggest that functional attributes being controlled for (spectral & temporal resolution, and sensitivity) likely play a critical for the robust coding of sounds.

The HSNN employs temporal coding and organizational principles identified physiologically and it can operate on multiple time-scales. Like the auditory pathway, the auditory HSNN is inherently temporal and is capable of processing detailed temporal fine structure modulations using nonlinear spiking neurons that can synchronize to fast acoustic features. As seen from the optimal HSNN receptive fields and observed physiologically (Fig 4), the early levels of the HSNN and auditory pathway operate on and encode fine temporal details and these transition to a coarser temporal representation. The deep layers of the network largely operate on relatively slow features in speech, such as consonant vowel transitions, syllable structure, and time-varying formant structure, which are known to be critical for recognition task [31,33,53]. The early levels of the optimal HSNN synchronize and preserves envelopes in speech exceeding several hundredths of Hz and this synchronization ability is sequentially degraded across layers (Fig 7C), analogous to changes in synchronization ability seen along the auditory pathway [5]. Such sequential transformations remove fine structure details (e.g., voicing periodicity) and noise and enhance the encoded speech signal, preserving relatively slow envelope cues that are critical to the recognition task, thus enhancing recognition accuracy in noise.

## Materials and methods

### Speech corpus

Sounds in the experimental dataset consist of isolated digits (*zero* to *nine*) from eight male talkers from LDC TI46 corpus [27]. Ten utterances for each digit are used for a total of 800 sounds (8 talkers x 10 digits/talker x 10 utterances/digit). Words are temporally aligned based on the waveform onset (first upward crossing that exceeds 2 SD of the background noise level) and speech babble noise (generated by adding 7 randomly selected speech segments) is added at multiple signal-to-noise ratios (SNR = -5, 0, 5, 10, 15 and 20 dB).

### Auditory model and hierarchical spiking neural network (HSNN)

All of the auditory model and HSNN simulations were performed using custom MATLAB software. We developed a multi-layer auditory network model consisting of a cochlear model stage containing gamma tone filters (0.1-4kHz; center frequencies 1/10^th^ octave separation; critical band resolution), envelope extraction and nonlinear compression [54] followed by a HSNN as illustrated in Fig 1. Several architectural and functional constraints are imposed on the spiking neural network to mirror auditory circuitry and physiology. First, the network contains six layers as there are six principal nuclei between the cochlea and cortex. Second, connections between consecutive layers contain both excitatory and inhibitory projections since long-range inhibitory projections between nuclei are pervasive in the ascending auditory system [12,55]. Each layer in the network contains 53 excitatory and 53 inhibitory frequency organized neurons per layer which allows for 1/10^th^ octave resolution over the frequency range of the cochlear model (0.1–4 kHz). Furthermore, since ascending projections in the central auditory pathway are spatially localized and frequency specific [20,55,56], excitatory and inhibitory connection weights are modeled by co-tuned Gaussian profiles of unspecified connectivity width (Fig 1**E**):

$$w_{l,m,n}^{E}=\frac{1}{\sqrt{2\pi \sigma_{E}^{2}}}{∙e}^{-{\left(x_{l,m}-x_{l+1,n}\right)}^{2}/2\sigma_{E}^{2}}$$ (Eq 2A)

$$w_{l,m,n}^{I}=\frac{1}{\sqrt{2\pi \sigma_{I}^{2}}}{∙e}^{-{\left(x_{l,m}-x_{l+1,n}\right)}^{2}/2\sigma_{I}^{2}}$$ (Eq 2B)

where $w_{l,m,n}^{I}$ and $w_{l,m,n}^{E}$ are the inhibitory and excitatory connection weights between the m-th and n-th neuron from layer *l* and *l*+1, *x*_*l*,*m*_ and *x*_*l*+1,*n*_ are the normalized spatial positions (0–1) along the frequency axis of the *m*-th and *n*-th neurons in layers *l* and *l*+1, and *σ*_*I*_ and *σ*_*E*_ are the inhibitory and excitatory connectivity widths (i.e., SD of Gaussian connection profiles), which determine the spatial spread and ultimately the frequency resolution of the ascending connections.

Each neuron in the network consists of a modified leaky integrate-and-fire (LIF) neuron [57] receiving excitatory and inhibitory presynaptic inputs (Fig 1**E**). Given a presynaptic spike trains from the *m*-th neurons in network layer-*l* (*s*_*l*,*m*_(*t*)) the desired intracellular voltage of the *n*-th neuron in network layer *l*+1 is obtained as

$$v_{l+1,n}(t)=\sum_{m}w_{l,m,n}^{E}∙h_{EPSP}(t)*s_{l,m}(t)-\beta \sum_{m}w_{l,m,n}^{I}∙h_{IPSP}(t)*s_{l,m}(t)$$ (Eq 3)

where * is the convolution operator, *β* is a weighting ratio between the injected excitatory and inhibitory currents, *h*_*EPSP*_(*t*) and *h*_*IPSP*_(*t*) are temporal kernels that model excitatory and inhibitory post synaptic potentials generated for each incoming spike as an alpha function (Fig 1**E**, red and blue curves) [57]. Since central auditory receptive fields often have extensive lateral inhibition/suppression beyond the central excitatory tuning area and inhibition is longer lasting and weaker [7,8] we require that *σ*_*I*_ = 1.5∙*σ*_*E*_, *τ*_*I*_ = 1.5∙*τ*_*E*_, and *β* = 2/3, as this produced realistic receptive field measurements. For simplicity, we use *σ* and *τ* interchangeably with *σ*_*E*_ and *τ*_*E*_, since these determine the overall spectral and temporal resolution of each neuron.

Because the input to an LIF neuron is a current injection, we derived the injected current that is necessary to generate the desired membrane voltage (from Eqn. 3). This was achieved by deconvolving the LIF neuron time-constant from the desired membrane voltage

$$i_{l+1,n}(t)=v_{l+1,n}(t)*h^{-1}(t)+z(t)$$ (Eq 4)

where *i*_*l*+1,*n*_(*t*) is the injected current for the *n*-th neuron in layer *l*+1 and *v*_*l*+1,*n*_(*t*) is the corresponding output voltage and *z*(*t*) is a noise current component. As we demonstrated previously [21], this procedure removes the influence of the cell membrane integration prior to injecting the current in the IF neuron compartment and allows us to precisely control the intracellular voltage delivered to each LIF neuron. Above $h(t)={\frac{1}{C}e}^{-t/\tau }u(t)$ is the impulse response of the cell membrane (*u*(*t*) is the step function), C is the membrane capacitance, *τ*, is the membrane time-constant and *h*^−1^(*t*) is the inverse kernel (i.e., *h*(*t*)*h^−1^(*t*) = *δ*(*t*) where *δ*(*t*) is the Diract function). Because the EPSP time constant and the resulting temporal resolution of the intracellular voltage are largely influenced by the cell membrane integration, we require that *τ* = *τ*_*E*_. Finally, Gaussian white noise, *z*(*t*), is added to the injected current in order to generate spike timing variability (signal-to-noise ratio = 15 dB) [21]. Upon injecting the current, the resulting intracellular voltage follows *v*_*l*+1,*n*_(*t*)+*z*(*t*)*h(*t*) and the IF model generates spikes whenever the intracellular voltage exceeds a normalized threshold value [21]. The normalized threshold is specified for each network layer (*l*) as

$$N_{l}=\left(V_{T}-V_{r}\right)/\sigma_{V,l}$$ (Eq 5)

where *V*_*T*_ = −45 mV is the threshold voltage, *V*_*r*_ = −65 mV is the membrane resting potentials, and *σ*_*V*,*l*_ is the standard deviation of the intracellular voltages for the population of neurons in layer *l*. As we have previously shown this normalized threshold represents the number of standard deviations the intracellular activity is away from the threshold activation and serves as a way of controlling the output sensitivity of each network layer. Upon generating a spike, the voltage is reset to the resting potential, a 1 ms refractory period is imposed, and the membrane temporal integration continues.

### Decision model

The neural outputs of the network consist of a spatio-temporal spiking pattern (e.g., Fig 1**G** and 1**H**, bottom panels), which is expressed as a *N*x*M* matrix **R** with elements *r*_*n*,*i*_ where *N* = 53 is the number of frequency organized output neurons and *M* is the number of time bins. The number of time bins is dependent on the temporal resolution for each bin, Δ*t*, which is varied between 0.5–100 ms. Each response (*r*_*n*,*i*_; *n*−th neuron and *i*−th time bin) is assigned a 1 or 0 value indicating the presence or absence of spikes, respectively.

A modified Bernoulli Naïve Bayes classifier [58] is used to read out the network spike trains and categorize individual speech words. The classified digit (*y*) is the one that maximizes posterior probability for a particular network response according to

$$y={\mathrm{argmax}}_{d=[0\cdots 9]}\prod_{n,i}p_{d,n,i}^{r_{n,i}}∙{\left(1-p_{d,n,i}\right)}^{1-r_{n,i}}$$ (Eq 6)

where *d* = 0 … 9 are the digits to be identified, *p*_*d*,*n*,*i*_ is the Bayesian likelihood, i.e. the probability that a particular digit, *d*, generates a spike (1) in a particular spatio-temporal bin (*n*-th neuron and *i*-th time bin). Note that the classifier estimates the total network posterior probability by accumulating the posterior probability from independent Bernoulli observations (individual time bins from individual neurons). When the response of a given time-neuron bin is 1, the contribution to the total posterior probability from that particular bin and neuron is precisely equal to the Bayesian likelihood (for that time-neuron bin and specific sound): $p_{d,n,i}^{1}∙{\left(1-p_{d,n,i}\right)}^{0}=p_{d,n,i}$. On the other hand, when the neuron generates 0 response (no spike), the contribution is the complement probability: $p_{d,n,i}^{0}∙{\left(1-p_{d,n,i}\right)}^{1}=1-p_{d,n,i}$. The total posterior probability for a given sound is accumulated over all possible time-neuron bins (*n*,*i*) under the assumption of independence. This procedure is repeated over all digits (*d*, by recomputing the posterior for each digit) and the decision is the digit (*d*) that maximizes the total posterior probability.

### Network constraints and optimization

The primary objective is to determine the spectral and temporal resolution of the network connections as well as the network sensitivity necessary for robust speech recognition. Specifically, we hypothesize that the temporal and spectral resolution and sensitivity of each network layer need to be hierarchically organized across network layers in order to maximize speech recognition performance in the presence of noise. We thus optimize three key parameters, the time constant (*τ*_*l*_), connectivity widths (*σ*_*l*_), and normalized threshold (*N*_*l*_) that separately control these functional attributes of the network, where the index *l* designates the network layer (1–6). Given that spectro-temporal selectivity changes systematically and gradually between auditory nuclei, we constrained the parameters to vary smoothly from layer-to-layer according to the power law rules of Eqn. 1. The initial parameters for the first network layer, *τ*_1_ = 0.4 ms, *σ*_1_ = 0.0269 (equivalent to ~1/6 octave), and *N*_1_ = 0.5 are selected to allow for high-temporal and spectral resolution and high firing rates, analogous to physiological characteristics of auditory nerve fibers [5,6,24,26] and inner hair cell ribbon synapse [25]. We optimize for the three scaling parameters *α_τ_*, *γ_σ_*, and *λ_N_*, which determine the direction and magnitude of layer-to-layer changes and ultimately the network organization rules for temporal and spectral resolution and network sensitivity.

The optimization is carried using a cross-validation grid search procedure in which we maximized word accuracy rates (WAR). Initial tests are performed to determine a suitable search range for the scaling parameters and a final global search is performed over the resulting search space (*α_τ_* = 0.9−2.3, *γ_σ_* = 0.8−1.5 and *λ_N_* = 0.5−1.6; 0.1 step size for all parameters). For each parameter combination, the network is required to identify the digits in the speech corpus with a ten-alternative forced choice task. For each iteration we select one utterance from the speech corpus (1 of 800) for validation and use the remaining utterances (799) to train the model by deriving the Bayesian likelihood functions (i.e., *p*_*d*,*n*,*i*_). The Bayesian classifier is then used to identify the validation utterances and compute WAR for that iteration (either 0 or 100% for each iteration). This procedure is iteratively repeated 800 times over all of the available utterances and the overall WAR is computed as the average over all iterations. This procedure is also repeated for five distinct signal-to-noise ratios (SNR = -5, 0, 5, 10, 20 dB). Example curves showing the WAR as a function of scaling parameters and SNR are shown in Fig 2 (**a** and **b**, shown for 5 and 20dB). The global optimal solution for the scaling parameters is obtained by averaging WAR across all SNRs and selecting the scaling parameter combinations that maximize the WAR (Fig 2**C**).

### Receptive field, mutual information, and response signal-to-noise ratio calculation

To characterize the layer-to-layer transformations performed by the network, we compute spectro-temporal receptive fields (STRFs) and measure the mutual information conveyed by each neuron in the network. First, STRFs are obtained by delivering dynamic moving ripple sounds (DMR), which are statistically unbiased, and cross-correlating the output spike trains of each neuron with the DMR spectro-temporal envelope [59]. For each STRF, we estimate the temporal and spectral resolution by computing the integration time and bandwidths, as described previously [7]. Mutual information is calculated by delivering a sequence of digits (0 to 9) at 5 dB SNR to the network. The procedure is repeated 50 trials with different noise seeds and the spike trains from each neuron are converted into a dot-raster sampled at 2 ms temporal resolution. The mutual information is calculated for each neuron in the network using the procedure of Strong et al. [60] as described previously [21].

Using the digit sequence (0 to 9) at 5 dB input SNR, we also computed the output SNR of each neuron at each layer of the network. For each individual artificial neuron, we first estimated the signal and noise spectrums. We assume an additive signal and noise model where the response of the *i*-th response trial of each neuron is, *r*_*i*_(*t*) = *s*(*t*)+*n*_*i*_(*t*) and the noise, *n*_*i*_(*t*), are independent across trials because the background speech babble was chosen from independent sound segments for each simulation trial. The average response is used to approximate the signal component of the response, $\hat{s}(t)=\langle r_{i}(t)\rangle$, where 〈∙〉 represents the average operator across trials. Using the estimated signal component, we then estimated the signal power spectrum (*P*_*ss*_(*f*)) by taking the Welch averaged periodogram of $\hat{s}(t)\;(P_{\hat{s}\hat{s}}(f)\approx P_{ss}(f)$),1024 sample Kaiser window; *β* = 5; equivalent frequency resolution of 2.5 Hz). Next, we estimated the signal + noise spectrum, *P*_*ss*_(*f*)+*P*_*nn*_(*f*), by computing the power spectrum of the individual trial responses, $P_{r_{i}r_{i}}(f)$, and subsequently averaging across trials, $\langle P_{r_{i}r_{i}}(f)\rangle \approx P_{\left(s+n)(s+n\right)}(f)=P_{ss}(f)+P_{nn}(f)$. For each neuron, the noise spectrum was then estimated by subtraction,

$$P_{\hat{n}\hat{n}}(f)=\langle P_{r_{i}r_{i}}(f)\rangle -P_{\hat{s}\hat{s}}(f)\approx P_{nn}(f)$$

and the output SNR was estimated as

$${SNR}_{output}(f)=10{log}_{10}\left(\frac{P_{\hat{s}\hat{s}}(f)}{P_{\hat{n}\hat{n}}(f)}\right).$$

Since speech envelopes can span several orders of magnitude in their temporal content and since different modulation frequency bands contribute to various aspects of speech perception, we measured and analyzed the output SNR and output modulation index of the network for different modulation frequency bands. We focused on the fluctuation / rhythm band (1–25 Hz) and the periodicity pitch (75–150 Hz) bands because of their perceptual relevance and because neurons at various level of the auditory pathway can synchronize to the temporal envelopes for these bands. The fluctuation band is particularly relevant for speech recognition and contains much of the low modulation frequency information content required for word identification [5,31,33]. By comparison, the periodicity pitch band encompasses the temporal fluctuations created by vocal fold vibration which can extend out to several hundred Hz [5,33]. Here we focused on the range 75–150 Hz because the periodicity for the male talkers analyzed falls within this range (see Fig 7). The band SNR is defined as

$${SNR}_{f_{1}-f_{2}}=\int_{f_{1}}^{f_{2}}{SNR}_{output}(f)df$$

where *f*_1_ and *f*_2_ are the lower and upper frequency of the selected band. Finally, we also computed the band modulation index defined as

$${MI}_{f_{1}-f_{2}}=\frac{\int_{f_{1}}^{f_{2}}P_{\hat{s}\hat{s}}(f)df}{P_{\hat{s}\hat{s}}(0)+P_{\hat{n}\hat{n}}(0)}.$$

Conceptually, the band modulation index corresponds to the signal power within the selected band normalized by the total response power at DC (0 Hz). It provides a measure of the strength of the modulation relative to the DC component of the response.

### Auditory system data

Previously published data from single neurons in the auditory nerve (*n* = 227) [26], auditory midbrain (Central Nucleus of the Inferior Colliculus, *n* = 125) [54], thalamus (Medial Geniculate Body, *n* = 88) and primary auditory cortex (*n* = 83) [8] is used to quantify transformations in spectral and temporal selectivity between successive auditory nuclei. Using the measured spectro-temporal receptive fields of each neuron (Fig 3), the spectral and temporal selectivity are quantified by computing integration times, response latencies, and bandwidths as described previously [7]. The integration time is a measure of the time window over which a neuron integrates the sound and, as we have previously demonstrated both analytically and empirically, it is inversely related to the maximum synchronization frequency of the neuron [7,45].

Sequential changes in selectivity across ascending auditory nuclei are summarized by comparing the neural integration parameters of each auditory structure (Fig 3**F**–3**H**). Since the auditory midbrain, thalamus and cortical data was limited to neurons above 1kHz and because auditory nerve data only contained 13 fibers (out of 227) with best frequency < 1kHz we only analyzed neural and HSNN units with best frequencies above 1kHz. This was done because low frequency neurons are biased for broader bandwidths (in octaves) than high frequency neurons [6,7,61] and the network itself follows a similar trend (as a result of the cochlear filters used).

### Generalized linear model (GLM) networks

To identify the role of linear and nonlinear receptive field transformations for noise robust coding, we developed two single-layers networks containing GLM neurons [34] (Fig 6**A**) that are designed to capture linear and nonlinear transformations of the HSNN.

First, we developed a single-layer LP (linear Poisson) network consisting of model neurons with linear spectro-temporal receptive fields followed by a Poisson spike train generator (Fig 6**A**). For each output of the optimal network (*m*-th output) we measured the STRF and fitted it to a Gabor model (*STRF*_*m*_(*t*,*f*_*k*_)) by minimizing the mean squared error between the measured STRF and the Gabor STRF model [45]. On average the fitted Gabor model accurately replicated the structure in the measured STRFs and on average accounted for 99% of the STRF variance (range 94–99.9%). The output firing rate of the *m*-th LP model neuron is obtained as

$$\lambda_{m}(t)=\lambda_{0}+G∙\sum_{k=1}^{N}S(t,f_{k})*{STRF}_{m}(t,f_{k})$$ (Eq 7)

where *S*(*t*,*f*_*k*_) is the cochlear model output, * is the convolution operator, *G* is a gain term, and *λ*_0_ is required to assure that the spike rates are positive valued and the firing maintains a linear relationship with the sound. *G* and *λ*_0_ were optimized by searching (grid search) for the average firing rate taken across all output neurons and sounds that matches the average firing rate (minimize average absolute difference) of the optimal HSNN network. The firing rate functions for each channel, *λ*_*m*_(*t*), are then passed through a nonhomogenous Poisson point process in order to generate the spike trains for each output channel.

We also explored the role of nonlinear rectification by incorporating a rectification stage in the LP model. The firing of the m-th neuron in the LNP (linear nonlinear Poisson) network is

$$\lambda_{m}(t)=G∙max[0,\sum_{k=1}^{N}S(t,f_{k})*{STRF}_{m}(t,f_{k})]$$ (Eq 8)

where the gain term, *G*, was optimized for so that the average firing rate taken across all output neurons and all words matches the average firing rate of the optimal HSNN (minimum average absolute difference).
